# Solution and on-surface synthesis of structurally defined graphene nanoribbons as a new family of semiconductors

**DOI:** 10.1039/c8sc03780a

**Published:** 2019-01-02

**Authors:** Akimitsu Narita, Zongping Chen, Qiang Chen, Klaus Müllen

**Affiliations:** a Max Planck Institute for Polymer Research , Ackermannweg 10 , D-55128 Mainz , Germany . Email: narita@mpip-mainz.mpg.de ; Email: muellen@mpip-mainz.mpg.de; b Institute of Physical Chemistry , Johannes Gutenberg-University Mainz , Duesbergweg 10-14 , D-55128 Mainz , Germany

## Abstract

Graphene nanoribbons (GNRs) are quasi-one-dimensional subunits of graphene and have open bandgaps in contrast to the zero-bandgap graphene. The high potential of GNRs as a new family of carbon-based semiconductors, *e.g.* for nanoelectronic and optoelectronic applications, has boosted the research attempts towards fabrication of GNRs. The predominant top-down methods such as lithographical patterning of graphene and unzipping of carbon nanotubes cannot prevent defect formation. In contrast, bottom-up chemical synthesis, starting from tailor-made molecular precursors, can achieve atomically precise GNRs. In this account, we summarize our recent research progress in the bottom-up synthesis of GNRs through three different methods, namely (1) in solution, (2) on-surface under ultrahigh vacuum (UHV) conditions, and (3) on-surface through chemical vapour deposition (CVD). The solution synthesis allows fabrication of long (>600 nm) and liquid-phase-processable GNRs that can also be functionalized at the edges. On the other hand, the on-surface synthesis under UHV enables formation of zigzag GNRs and *in situ* visualization of their chemical structures by atomic-resolution scanning probe microscopy. While the on-surface synthesis under UHV is typically costly and has limited scalability, the industrially viable CVD method can allow lower-cost production of large GNR films. We compare the three methods in terms of the affordable GNR structures and the resulting control of their electronic and optical properties together with post-processing for device integration. Further, we provide our views on future perspectives in the field of bottom-up GNRs.

## Abstract 2

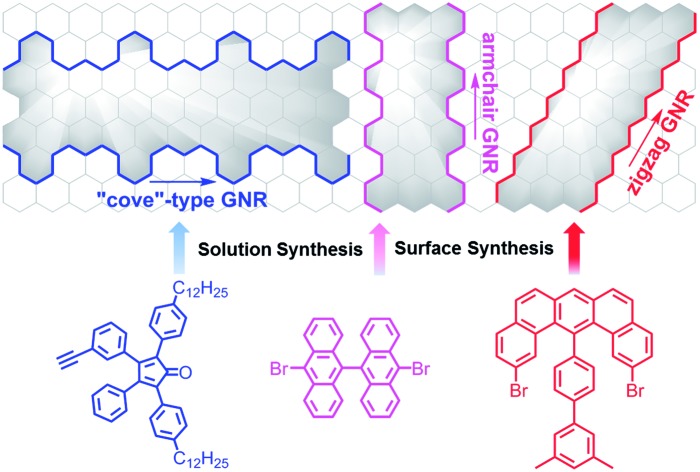
Graphene nanoribbons (GNRs) with various structures and properties can be synthesized in solution or on surface.

## 1. Introduction

Amongst the many characteristics of the 2D-material graphene, its high charge-carrier mobility is one of the most important since it gives graphene a promise of excellent performance in field effect transistors (FETs).^1^–^5^ However, this advantage is diminished by the vanishing bandgap of graphene, which prohibits on/off switching of the current through the device.^6^–^14^ Therefore, unless the application calls for a switch that is never “off”, a bandgap opening is required, and one way to achieve that is a geometric confinement like what is seen in graphene nanoribbons (GNRs).^9^–^18^ A GNR can thus utilize the electronic advantages of the honeycomb graphene structure and offers a controlled, finite bandgap to generate a new family of carbon-based semiconductor materials.

A GNR can be regarded as a quasi-one-dimensional graphene cut-out, and this makes it an analogue of a large class of conjugated polymers for which optimization of the synthesis and method of film formation are the keys to device fabrication.^12^,^19^,^20^ The structural perfection of a GNR is indeed an essential issue because its electronic and optical properties are critically dependent on its chemical structure, namely, its edge configuration, width and crystal direction.^17^,^21^,^22^ GNRs with armchair and zigzag edges are the most representative cases. Armchair GNRs (AGNRs) possess a wide range of bandgaps that vary with the width, while zigzag GNRs (ZGNRs) are predicted to have smaller bandgaps with localized edge states that are magnetic and show great potential for spintronic applications.^23^–^28^

In view of their promise in FET applications as well as the intriguing structure–property relationship of GNRs, it is not surprising that physicists, chemists, materials scientists and electrical engineers have made numerous attempts at GNR fabrication.^8^–^12^,^14^,^18^,^19^,^21^,^22^,^29^,^30^ These syntheses have mainly been *via* so-called top-down methods, as represented by the “cutting” of graphene through various lithographical methods using for example an electron beam,^31^ a helium ion beam,^32^,^33^ a scanning tunnelling microscope,^34^ and plasma etching with a metal nanowire mask^35^ or *via* sonochemical extraction.^16^ Another typical top-down method is the “unzipping” of carbon nanotubes, which has been accomplished, for instance, by chemical oxidation^36^ and plasma etching.^37^ GNRs of different lengths, down to <5 nm, have been prepared by such methods and have shown promising electronic properties, such as high on-off ratios (up to 10^6^), that make them applicable in devices and other future applications.^38^ Nevertheless, most GNRs prepared by top-down methods have undefined edge structures, and the above methods have thus far failed to accurately control the resulting GNR structures on the atomic scale. Instead, bottom-up chemical syntheses, starting from small-molecule precursors, have emerged as reliable methods to reproducibly provide GNRs with precise atomic control.^11^,^21^,^22^,^39^

Bottom-up methods consist of two main pathways, namely, (1) in solution using synthetic organic and polymer chemistry techniques, and (2) on substrates with modern surface science techniques.^21^,^22^,^29^,^39^ Representative solution synthesis methods have extended three-dimensional polyphenylenes as precursors and modify them *via* intramolecular oxidative cyclodehydrogenation, namely, the Scholl reaction, typically with FeCl_3_ serving as both the oxidant and Lewis acid.^40^–^43^ The topology of such polyphenylene precursors, which consist of multiple benzene rings connected with single bonds, must be specifically designed so that the “planarization”, in this case, “graphitization”, into the GNRs proceeds without defects. Alternatively, the final C–C bond formation between the benzene rings can also be performed through photochemical cyclodehydrochlorination reaction, by selectively introducing chloro groups in polyphenylene precursors.^44^,^45^ Acid-promoted benzannulation of alkynes has also proved to be highly useful to this end, enabling a synthesis of *N* = 5 armchair GNR with the width of five carbon atoms from a poly(2,6-dialkynyl-*p*-phenylene) as the precursor.^46^,^47^

In 2010, metal-surface-assisted coupling of aryl halides was applied to the preparation of polyphenylene precursors of GNRs, and subsequent cyclodehydrogenation led to the direct formation of GNRs on the surface.^48^ The resulting GNRs could be visualized *in situ* by high-resolution scanning tunnelling microscopy (STM) under ultrahigh vacuum (UHV) conditions, unambiguously proving their atomically precise structures. During the last decade, both in-solution and on-surface GNR syntheses have advanced in a highly complementary manner and provided a variety of GNRs with different widths, edge structures, and heteroatom substituents in the aromatic core.^22^,^39^,^43^,^46^,^49^–^62^ Above all, these carefully designed techniques have allowed the fine tuning of their electronic and optical properties.

Solution syntheses can be scaled up to the gram scale and have also furnished GNRs that are longer than 100 nm on average and dispersible in organic solvents for characterizations in and processing from the liquid phase.^43^,^63^ The edges of the GNRs can be decorated with alkyl chains to enhance the dispersibility^43^ or with different functional groups, which can, for example, affect the electronic properties and self-assembly behaviour of the GNRs.^64^ Remarkably enough, edge functionalization with organic radicals has led to the first experimental demonstration of long-sought-after GNRs with magnetic edge states and pronounced chemical stability.^65^

The size and structural complexity of such large conjugated macromolecules present new challenges for characterization, and quite naturally, the structural perfection of many such solution-synthesized GNRs remains an open question. It is therefore important that the on-surface synthesis and characterization of GNRs can reveal the formation of desired GNR structures and also accurately indicate the presence or absence of defects. Furthermore, the UHV environment enables the formation of unstable structures that are incompatible with the solution method.^21^ Indeed, ZGNRs have become accessible, and their localized edge states, as theoretically predicted, can be verified.^43^ The relatively high cost and limited scalability of on-surface syntheses using the UHV conditions are clearly problematic for practical applications, but this issue has recently been addressed by the development of a modified method employing an industrially viable chemical vapour deposition (CVD) strategy.^66^,^67^ A film of GNRs could thus be grown over larger areas and under low vacuum or even at an ambient pressure, which represents a step towards the technological application of such bottom-up syntheses of GNRs. In this account, we briefly summarize the recent progress in our group on the bottom-up synthesis of GNRs through solution-mediated and surface-assisted methods. We also discuss the future challenges and perspectives. Readers are advised to refer to previous review articles by others^30^,^68^,^69^ and by ourselves^22^,^39^,^42^,^70^–^73^ for more comprehensive descriptions of previous works in the field.

## 2. Solution-mediated synthesis of GNRs

**Fig. 1:**
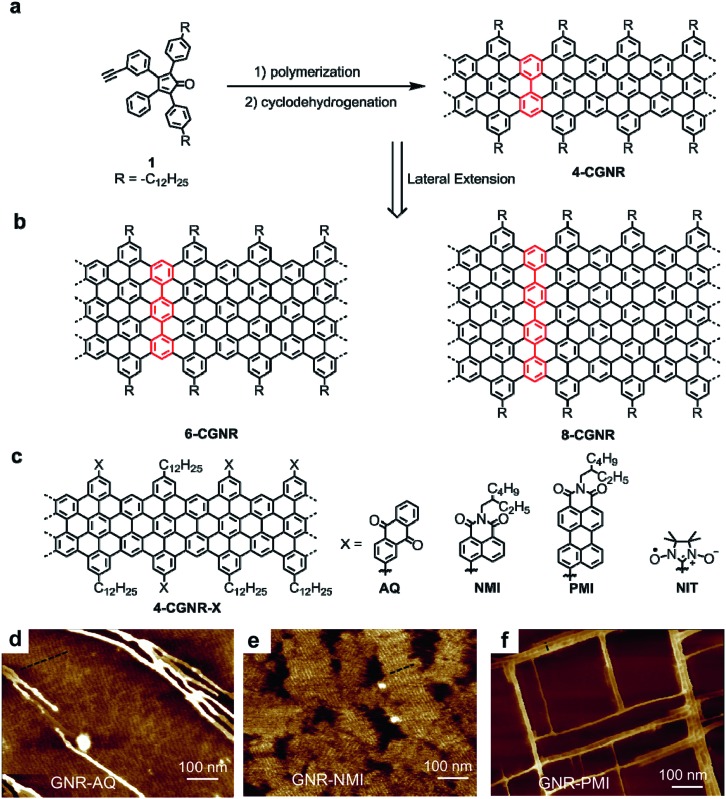
(a) Synthesis of **4-CGNR** by Diels–Alder polymerization of A–B type monomer **1** and cyclodehydrogenation. (b) Structures of laterally extended **6-CGNR** and **8-CGNR**. (c) Structures of edge-functionalized **4-CGNR-X** with anthraquinone (AQ), naphthalene monoimide (NMI), perylene monoimide (PMI) and nitronyl-nitroxide radical (NIT) groups. (d–f) AFM images of **4-CGNR-AQ**, **4-CGNR-NMI** and **4-CGNR-PMI** drop-casted on graphite in 1,2,4-trichlorobenzene solvent and annealed at 100 °C. Reproduced with permission.^64^ Copyright (2017) American Chemical Society.

Approximately ten years ago, polyphenylene precursors for GNRs were prepared through a palladium-catalysed A_2_B_2_-type Suzuki polymerization^40^,^74^ involving two different monomers and *via* a nickel-mediated AA-type Yamamoto polymerization of single dihalogenated monomers.^75^,^76^ However, these polycondensation methods unavoidably involve expensive transition-metal catalysts/reagents, which are also difficult to remove after the reaction. Moreover, these methods result in polymers with relatively low molecular weights and, in most cases, lead to GNRs shorter than 50 nm. Therefore, we recently turned to AB-type Diels–Alder cycloadditions as a catalyst-free and highly efficient reaction to prepare polyphenylenes.^43^ By this method, monomer **1** with a tetraphenylcyclopentadienone core as the diene and an ethynyl group as the dienophile (Fig. 1a) served as the key starting material. The polymerization of **1** proceeded simply by heating in a diphenyl ether solution in the absence of any catalyst or reagent and gave polyphenylene precursors with extremely high weight-average molecular weights of >600 000 g mol^–1^. Molecular weight determinations of rigid polymers can be challenging, but these remarkable values were confirmed by laser-light-scattering experiments. Subsequent cyclodehydrogenation led to GNRs with “cove”-type edges with widths of four sp^2^ carbon atoms at their narrowest point (hereafter called **4-CGNR**) and lengths greater than 600 nm.^43^,^77^ **4-CGNR** was then employed in numerous attempts at the fabrication of single-GNRs and thin-film FET devices.^78^ The observed device performance indicated charge-carrier mobilities much lower than the intrinsic data indicated by time-resolved terahertz (THz) photoconductivity measurements and theoretical calculations.^79^ We assumed that the device performances were compromised by the large bandgap of **4-CGNR** and high contact resistance in addition to other possible factors such as aggregation, conformational defects and the insulating alkyl chains wrapping around the GNRs. Thus, we next focused on the synthesis of lower-bandgap GNRs while maintaining a length of >100 nm.

A straightforward method to reduce the bandgap is to make the GNR wider. The notable advantage of the bottom-up synthesis method is that the final GNR structure is uniquely dependent on the monomer structure, which can be changed at will within the capabilities of the modern synthetic chemistry. Lateral extension of **4-CGNR** was thus carried out by careful modification of the monomer structure, namely, by adding additional phenyl groups to the peripheral rings, followed by a similar procedure of the AB-type Diels–Alder polymerization and oxidative cyclodehydrogenation. This modification provided wider GNRs, **6-CGNR** and **8-CGNR**, with widths of six and eight sp^2^ carbon atoms, respectively, at their narrowest (Fig. 1b).^49^,^79^,^80^ **6-CGNR** and **8-CGNR** displayed electron absorption spectra that extended into the near-infrared (NIR) region and lowered optical bandgaps of approximately 1.4 and 1.2 eV, respectively, compared with approximately 1.9 eV for **4-CGNR**. However, the extended aromatic core of the wider GNRs compromised their dispersibility and processability, which initially prohibited, for example, the realization of higher charge-carrier mobilities in thin-film FET devices, as expected for the smaller bandgaps.^49^,^79^ The decreased processability of the wider GNRs prompted us to develop an alternative strategy for reducing the bandgap of the GNRs that did not rely on lateral extension.

In 2013, we addressed the synthesis of n-type GNRs, which are remarkably underdeveloped, through chlorination of the GNR edges by treatment with AlCl_3_ and ICl in CCl_4_.^81^ Unexpectedly, we observed that the bandgap of the GNRs decreased upon edge chlorination without changing the structure of the aromatic core, and this decrease was supported by density functional theory (DFT) calculations. The chlorinated GNRs could not be integrated into FET devices due to their low dispersibility, because of the absence of solubilizing alkyl chains. More recently, however, we developed a general strategy for the edge substitution of GNRs based on the Suzuki coupling of polyphenylene precursors bearing both long alkyl chains and bromo groups prior to the cyclodehydrogenation.^64^ As initial examples, we chose several electron-deficient groups, namely, anthraquinone (AQ) and naphthalene/perylene monoimide (NMI/PMI), as substituents to assess the feasibility of (1) lowering the bandgap and (2) obtaining n-type GNRs without compromising the processability. Based on DFT calculations, the edge-substituted GNRs, **4-CGNR-AQ**, **4-CGNR-NMI** and **4-CGNR-PMI**, were predicated to have n-type character with lowered conduction and valence band energy levels, and **4-CGNR-AQ** was calculated to have a bandgap (1.69 eV) considerably lower than that of pristine **4-CGNR** (2.05 eV). However, the changes in the bandgap of the substituted **4-CGNRs** could not be directly determined from their optical absorption spectra, which did not significantly change with the edge substitution. This observation was consistent with the theoretical results obtained from the time-dependent DFT (TD-DFT) calculations, which revealed that the differences in the electronic bandgaps were not directly reflected in the optical spectra. Nevertheless, these substituted **4-CGNRs** showed liquid-phase processability comparable to the pristine **4-CGNRs** with only alkyl chains and allowed the facile formation of self-assembled films on graphite surfaces. To our surprise, an investigation of such films on graphite by atomic force microscopy (AFM) revealed that the identity of the substituents on the edges affects the self-assembly behaviour of the GNRs. While **4-CGNR-AQ** and **4-CGNR-NMI** displayed domains of aligned GNRs, **4-CGNR-PMI** demonstrated unique rectangular networks of bundles of GNRs, which had never been observed for other GNR structures (Fig. 1d–f). We assume that this “crossing” of the GNRs at 90° was induced by the interactions between the GNR cores and the extended aromatic cores of the pendant PMI units (Fig. 1c).^64^ On the other hand, by utilizing **4-CGNR-Br** bearing bromo groups, we also succeeded in introducing nitronyl-nitroxide radicals (NIT) on the edges of the GNRs.^65^ Studies by electron spin resonance (ESR) spectroscopy and spin density calculations revealed spin injection from the NIT radical units into the GNR core, providing the first experimental demonstration of the long-sought-after magnetic edge states of GNRs. **4-CGNR-NIT** shows relatively high stability and can be obtained as a powder, unlike the unstable ZGNRs that were formed on metal surfaces under UHV.^21^ We thus obtained a promising alternative for the important potential spintronic applications of GNRs.^82^–^88^ Moreover, interactions between spins localized on the NIT units and the spins of the edge states of **4-CGNR-NIT** were demonstrated, which paves a way towards the realization of GNR-based logic gates for quantum computers.

## 3. On-surface synthesis of GNRs under UHV conditions

**Fig. 2:**
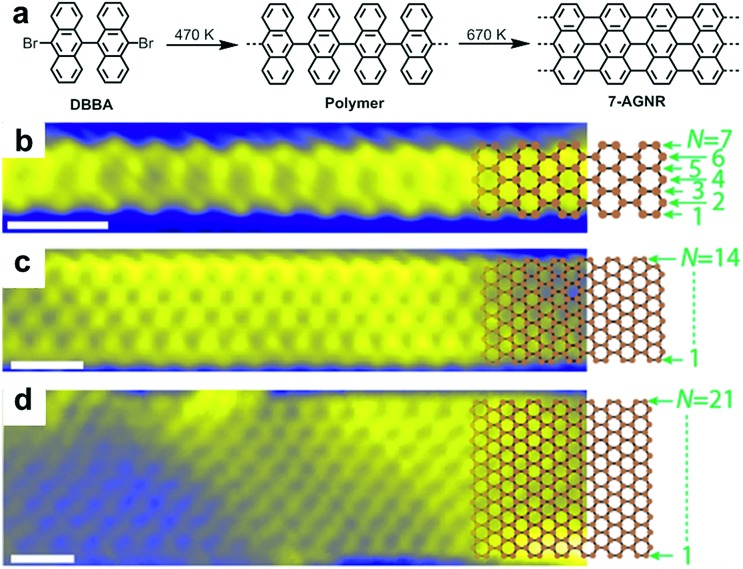
Bottom-up synthesis of atomically precise AGNR from 10,10′-dibromo-9,9′-bianthryl (**DBBA**) monomer. (a) Synthetic route toward **7-AGNR**. (b–d) STM images of **7-**, **14-**, and **21-AGNR** with partially superimposed sp^2^ carbon framework structures. Reproduced with permission.^97^ Copyright (2017) American Chemical Society.

In close collaboration with the group of Roman Fasel, who has been a pioneer in this field, we produced our first report on the surface-assisted synthesis of GNRs in 2010.^48^ This paper demonstrated the formation and direct STM visualization of atomically precise, straight *N* = 7 AGNR (**7-AGNR**), which is seven carbon atoms wide, as well as so-called chevron-type GNRs (see Fig. 2a for the structure). These materials were synthesized through thermally induced polymerization of 10,10′-dibromo-9,9′-bianthryl (**DBBA**) and 6,11-dibromo-1,2,3,4-tetraphenyltriphenylene, respectively, as monomers on a Au(111) surface under UHV conditions followed by cyclodehydrogenation at higher temperatures. The mechanism of the polymerization of the dibromo monomers on the Au(111) surface is supposed to involve the homolytic cleavage of the C–Br bonds to generate diradical intermediates, which then undergo polymerization, possibly involving formation of C–Au bonds.^89^–^91^ Notably, the diradical species can move across the surface without quenching, most likely because of the stabilization from the metal surface, which might be in a form of “temporal” complexation to the surface metal atoms, and thanks to the absence of solvent, which is a prerequisite for standard solution chemistry. The success of the reaction may be related to the orthogonal arrangement of the two anthryl moieties, which could lift the radical moieties off the surface of the metal to prevent complete immobilisation of the diradical species. Nevertheless, the reaction is not ideal as partial cyclodehydrogenation can occur at the polymerization temperature, and this reaction releases hydrogen atoms, which can terminate the polymerization.^92^ Thus, because of this termination pathway and due to possible contamination of the monomer with the monobromo species, it is not surprising that GNRs synthesized by on-surface methods are very often short (<50 nm).^93^,^94^ Visualization of the resulting **7-AGNRs** by atomic-resolution STM and noncontact-AFM (nc-AFM) with a tip functionalized with a carbon monoxide molecule revealed the presence of atomically precise structures with no defect.^95^,^96^ Although structures with defects, such as missing benzene rings and branching of GNRs due to the coupling of the end of a GNR to the edge of another ribbon, can be observed, all of these “errors” can be fully elucidated by nc-AFM, which for example can reveal the formation of a seven-membered ring at the branching site of two GNRs.^95^

After our first report, a number of research groups successfully replicated the on-surface synthesis of these two GNRs and reported a wide range of further physical characterization data using various spectroscopic and STM/AFM-based methods^99^–^104^ and demonstrated derivatization of the monomers to achieve new GNRs with modified structures and properties.^55^,^56^,^105^–^110^ It was also revealed that two or more GNRs can undergo lateral fusion to generate wider GNRs, for example, **14-AGNR** and **21-AGNR** from **7-AGNRs**,^93^,^97^ allowing the investigation of the electronic properties of GNRs with different widths by local scanning tunnelling spectroscopy (STS) measurements (Fig. 2).^98^

**Fig. 3:**
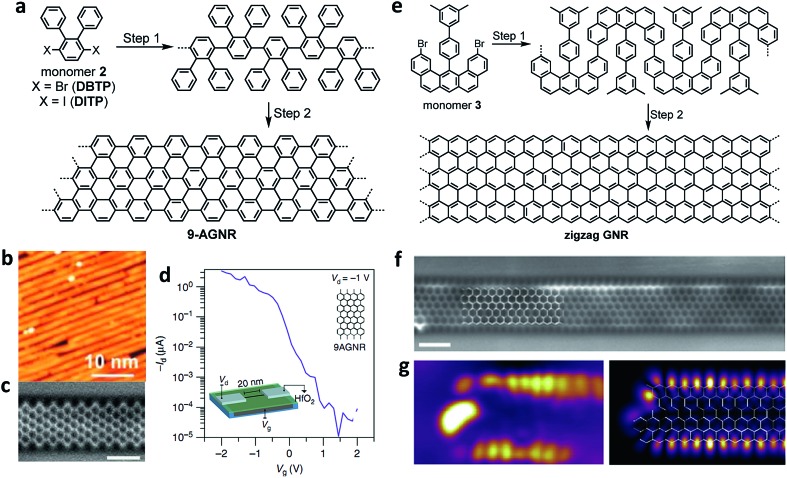
On-surface syntheses of GNRs under UHV conditions. (a) Synthetic route towards **9-AGNR** from monomer **2**. (b) STM images showing the length of the **9-AGNR** obtained from **DITP**. Reproduced with permission.^94^ Copyright (2018) American Chemical Society. (c) High-resolution noncontact atomic force microscopy (nc-AFM) frequency shift image of **9-AGNR** using a CO-functionalized tip. Reproduced with permission.^111^ Copyright (2017) American Chemical Society. (d) *I*_d_–*V*_g_ of the FET devices showing high *I*_on_ (>1 μA) for a 0.95 nm wide **9-AGNR** and high *I*_on_/*I*_off_ (∼10^5^). Inset: scaled **9-AGNR** FET schematic. (e) Synthetic route towards a full zigzag GNR from umbrella-shaped monomer **3**. (f) Constant height nc-AFM frequency shift image of ZGNR taken with a CO-functionalized tip. (g) Differential conductance maps of filled edge states taken at a sample bias of –0.3 V (left), and density functional theory (DFT)-based local density of states (DOS) at a tip-sample distance of 4 Å, showing the spatial distribution of filled edge states (right). All scale bars are 1 nm. (f and g) Reproduced with permission.^21^ Copyright 2016, Macmillan Publishers Ltd.

Stimulated by this ever growing interest, we have continued our tight collaboration with the group of Roman Fasel to develop novel design concepts for the synthesis of an increasing range of GNRs, which has allowed us to produce AGNRs with different widths as well as cove-edge GNRs and ZGNRs.^21^,^111^,^112^ For example, after multiple unsuccessful attempts with different monomer designs, we have finally achieved a synthesis of *N* = 9 AGNR with a width of 9 carbon atoms (**9-AGNR**) by employing 3′,6′-dibromo/diiodo-*ortho*-terphenyl (**2**) (**DBTP**/**DITP**) as the monomer precursor and demonstrated their atomically precise structures by high-resolution STM and nc-AFM (Fig. 3a–c).^94^,^111^ In particular, the use of **DITP** as the monomer allowed the growth of **9-AGNRs** (average length of approximately 45 nm) that were longer than those prepared from **DBTP** (average length of approximately 15 nm).^94^,^111^ This result is most likely because the lower polymerization temperature that could be used with **DITP**, possessing iodo groups, suppressed the concomitant cyclodehydrogenation. Hydrogen released by such simultaneous cyclodehydrogenation can most probably terminate the polymerization, leading to shorter GNRs (see the discussion above). STS measurements revealed a bandgap of 1.4 eV for **9-AGNR**, which is much lower than that of **7-AGNR** (2.4 eV), and this is in accordance with the theoretical values. The initial device studies using on-surface-synthesized **7-AGNR** by Bokor and co-workers reported compromised FET performance, which was attributed to the large bandgap and was similar to what was seen with the solution-synthesized **4-CGNR** discussed above.^100^ In contrast, FET studies conducted in collaboration with Fasel and Bokor using **9-AGNR** with the lower bandgap exhibited significantly improved performance with high on-current of 1 μA at a drain voltage of –1 V and a remarkable on-off ratio up to 10^5^ (Fig. 3d).^113^

After the synthesis of AGNRs in 2010, one of the most important challenges facing the field was the synthesis of ZGNRs, which had been predicted to display magnetic edge states localized along the zigzag edges. The difficulty in their synthesis stemmed from the fact that the zigzag edge could not be formed through conventional on-surface reactions, which only allowed C–C bond formation between benzene rings, giving an armchair edge. To this end, with the group of Roman Fasel, we designed umbrella-shaped monomers **3** with a pre-installed partial zigzag edge as well as methyl groups that were expected to undergo oxidative cyclization with the neighbouring aromatic rings to form extended zigzag edges (Fig. 3e).^21^ The synthesis of monomer **3** itself was highly challenging but could be achieved in 14 total steps involving platinum-catalysed cycloaromatization of the ethynyl groups to form the zigzag edge. Monomer **3** allowed the fabrication of the long-awaited ZGNRs with atomically precise structures on a Au(111) surface as visualized by nc-AFM (Fig. 3e–g).^21^ The theoretically predicted existence of localized edge states with a large energy splitting was experimentally confirmed by STS measurements close to the zigzag edges (Fig. 3g). Further characterizations of the ZGNRs, and in particular, the investigation of the predicted magnetic properties of the edge states, will be the next challenge and will require, for example, the application of non-conventional STM with spin-polarized tips or the development of a method to perform ESR spectroscopy *in situ* on the gold surface.

## 4. On-surface synthesis of GNRs through CVD

**Fig. 4:**
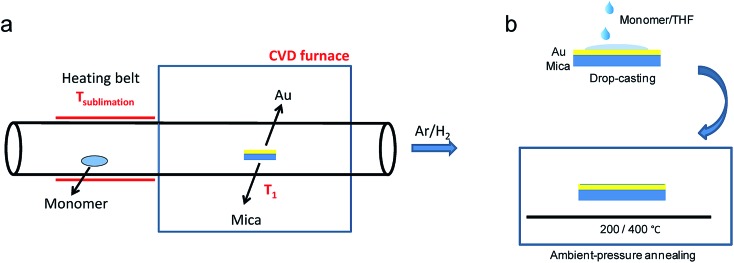
Schematic illustration comparing GNR synthesis through (a) CVD and (b) solution processing, which avoids the use of heat and vacuum for the monomer deposition. (a) Reproduced with permission.^66^ Copyright (2016) American Chemical Society; (b) Reproduced with permission.^120^ Copyright (2017) The Chemical Society of Japan.

**Fig. 5:**
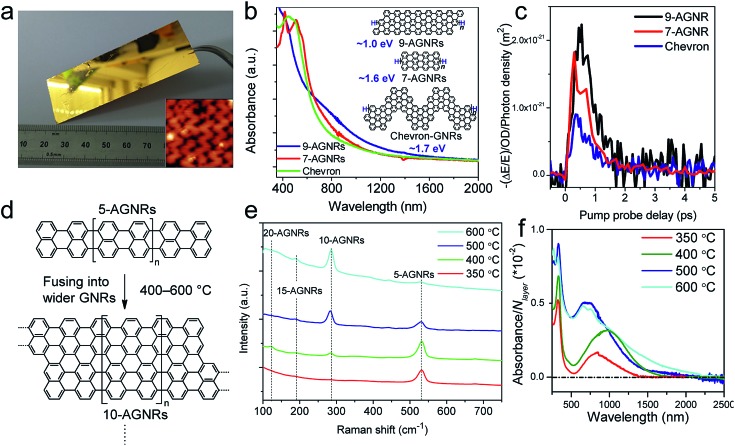
On-surface synthesis of GNRs by CVD. (a) Photograph of a 25 × 75 mm^2^ Au/mica plate on which a GNR film could be grown. Inset shows the STM image of chevron-type GNRs. Reproduced with permission.^66^ Copyright (2016) American Chemical Society. (b) UV-vis-NIR absorption spectra of different CVD-grown GNRs. The inset shows the chemical structures of the GNRs and their optical bandgaps. (c) Comparative study of THz photoconductivity of different GNR structures. (b and c) Reprinted with permission.^115^ Copyright (2017) American Chemical Society. (d) Lateral fusion of **5-AGNR** to form wider GNRs. (e) Raman spectra of extended AGNRs annealed at different temperatures showing the RBLM peaks of various GNRs of different widths. (f) UV-vis-NIR absorption spectra of AGNRs annealed at different temperatures. The spectra are normalized to the number of GNR layers (N layer). (d–f) Reprinted with permission.^116^ Copyright (2017) American Chemical Society.

While the on-surface syntheses under UHV allow the formation and visualization of atomically precise GNRs with various structures and properties, the amount of GNRs produced is limited to a monolayer over areas smaller than 1 cm^2^. For future applications of GNRs, a facile and scalable alternative to on-surface synthesis methods that does not require rigorous UHV conditions must be developed. The latter approach requires expensive equipment that generally includes a very small reaction chamber, limiting the scalability of the synthesis. The UHV conditions are necessary for the high-resolution visualizations by STM and AFM and to suppress possible side reactions of the diradical intermediates, polymers and/or resulting GNRs with oxygen, water and other atmospheric contaminants. Nevertheless, UHV is not essential for the on-surface reaction itself, which can potentially also be carried out under less demanding high vacuum (HV) conditions^114^ or even under atmospheric pressure. Typical setups for CVD were utilized to test a similar on-surface GNR synthesis through (1) sublimation of the same monomer precursors as used in the UHV protocol, (2) deposition on a gold surface inside a horizontal tube furnace, and then (3) thermal annealing to induce surface-assisted polymerization and cyclodehydrogenation (see Fig. 4a). This CVD method indeed allowed the synthesis of different AGNRs as well as chevron-type GNRs over large areas (>18 cm^2^) even at atmospheric pressure under argon/hydrogen (Fig. 5a and b),^66^,^115^–^117^ and this synthesis was also demonstrated by Nakae, Sakaguchi, and their colleagues.^67^,^118^ The increased availability of the GNR films allowed optoelectronic characterization of the CVD-grown GNRs by transferring multiple layers onto a transparent substrate. For example, broad optical absorption extending to ∼1200 nm was observed for the multilayer film of **9-AGNR**, suggesting an optical bandgap of ∼1.0 eV, which was significantly smaller than those of **7-AGNR** (∼1.6 eV) and the chevron-type GNR (∼1.7 eV) (Fig. 5b).^115^ Time-resolved THz spectroscopy measurements revealed that **9-AGNR** had the highest photoconductivity amongst these three GNRs and an intrinsic charge-carrier mobility of approximately 350 cm^2^ V^–1^ s^–1^ (Fig. 5c). We have also recently demonstrated the highly efficient lateral fusion of **5-AGNR** into wider AGNRs, including **10-** and **15-AGNRs**, at higher temperatures (Fig. 5d).^116^ The lateral fusion was confirmed by the appearance of new radial breathing-like mode (RBLM) peaks in the Raman spectra at 285, 188, and 122 cm^–1^ (Fig. 5e). These peaks are indicative of the width and are in excellent agreement with the DFT-calculated RBLM peaks for **10-**, **15-**, and **20-AGNRs**, respectively.^119^ UV-vis-NIR absorption spectroscopy revealed distinct absorption profiles for samples annealed at different temperatures, indicating highly efficient fusion of the GNRs in contrast to the local formation of wider GNR segments observed under UHV conditions. The presence of **10-AGNR** was observed after heating to 500 °C (Fig. 5f). The GNRs treated at 600 °C showed very broad absorptions extending into the infrared region up to ∼2250 nm, indicating such GNRs have potential for infrared sensing applications.

The CVD method still involves thermal and/or vacuum sublimation of monomer precursors, which consumes large amounts of energy and precludes the use of thermally unstable and/or large monomers that cannot be sublimed. To address this problem, we developed a new on-surface method for the synthesis of GNRs through solution processing of precursors on the same gold surface without sublimation followed by thermally induced polymerization and cyclodehydrogenation (Fig. 4b).^120^ Raman spectra confirmed the high quality and uniformity of the resulting GNRs, which were comparable to those fabricated by the CVD and UHV methods. This on-surface synthesis *via* solution processing is expected to allow straightforward scale-up and cost reductions that could be key to the wider application of bottom-up-synthesized GNRs.

## 5. Conclusions and outlook

There have been significant advancements in the bottom-up synthesis of GNRs over the last decade, and a wide range of structures and properties are attainable. Solution-mediated and surface-assisted methods have been developed in parallel and in a highly complementary manner. While the bulk-scale synthesis of GNRs has been achieved in solution with the potential for edge functionalization and liquid-phase processing,^43^,^79^ cleanly depositing such solution-synthesized GNRs on insulating substrates for the fabrication of well-functioning devices remains challenging. In contrast, the on-surface synthesis allows the preparation of flat GNR films that can be studied *in situ* by STM and AFM and transferred onto arbitrary substrates for further characterization and device integrations. The use of a metal surface and UHV conditions even allows the synthesis and direct visualization of pristine ZGNRs. On the other hand, solution synthesis of the same ZGNR appears to be impossible, considering the structural analogy to polyacenes^121^–^126^ and higher periacenes,^127^,^128^ which can be regarded as very short 4-ZGNRs four carbon atoms wide. The synthesis should be extremely challenging even with kinetic protection by multiple bulky substituents. The on-surface synthesis was recently used to produce GNRs with topological quantum states, opening up the potential of GNRs as new topological materials.^129^,^130^

Nevertheless, substitution of the edges with functional groups remains challenging using surface-assisted synthesis, and thus solution-mediated synthesis is important to this end. Edge substitution with different anchor groups is key to the further development of GNRs to achieve (1) programmed self-assembly of GNRs; (2) tailored optical and electronic properties; (3) magnetic properties; and (4) other functions such as stimuli responsiveness, water solubility and coordination of metal ions, *e.g.*, for catalytic activity. In particular, substitution of GNR edges with spin-bearing groups has emerged as a promising method for preparing GNRs with magnetic edge states for spintronic applications without requiring zigzag edges, and the edge states and appended spins can potentially be used as qubits for supercomputing applications.^65^

On the other hand, the surface-assisted synthesis allows reliable and precise heteroatom substitution of the GNR edges, which is often difficult by solution synthesis due to the lower stability and reactivity of heteroatom-substituted precursors under the Lewis acidic and oxidative conditions of the cyclodehydrogenation reaction. Heteroatom substitution can modulate the electronic properties and can also lead to networks of GNRs with controllable self-assembly behaviour, for example by utilizing the hydrogen bonding previously demonstrated for nitrogen-doped GNRs.^51^,^54^,^55^,^107^

For thin-film electronics, the on-surface-synthesized GNRs currently provide more promising results than are observed with solution-synthesized GNRs. This difference is presumably due to the flat deposition of the GNRs directly prepared on the surface, which circumvents the aggregation-related problems of their solution-synthesized counterparts. In particular, the CVD method and the protocol involving the solution processing of monomers have successfully allowed the facile large-area synthesis and transfer of GNRs for device integration.^66^ Moreover, the remarkable optical properties of the CVD-synthesized low-bandgap GNR films with absorptions over the visible and infrared regions give them great potential for optoelectronic applications such as in photovoltaics and visible-to-infrared sensors.^115^ The fabrication of FET devices based on single isolated GNRs is of great interest for fully utilizing the intrinsic properties of the GNRs, which can be more promising with GNRs prepared by solution synthesis or on surfaces under UHV conditions rather than the CVD method. If a silicon-based complementary metal-oxide-semiconductor (CMOS) technology will come to an end is unclear, but GNRs as a new family of semiconductor materials can offer different advantages, for example, in terms of miniaturization and theoretical FET performances that exceed those of the silicon-based CMOS^131^ as well as potential for single-electron transistors. Carbon nanotubes as another promising carbon-based semiconductor material have inherent problem associated with the challenging chirality-selective synthesis and contamination by metallic species.^132^–^137^ In contrast, GNRs can be readily prepared by bottom-up strategies with uniformly designed structures and purely semiconducting properties with pre-defined bandgaps. The continuous and growing overlap amongst chemistry and physics, surface science, materials science, and electrical engineering is essential for the further development of the field of GNRs in terms of realizing new structures, elucidating their emerging physical properties, achieving proof of concepts in devices, and future applications.

## Conflicts of interest

There are no conflicts of interest to declare.
